# NRF2 Deficiency Attenuates Diabetic Kidney Disease in Db/Db Mice via Down-Regulation of Angiotensinogen, SGLT2, CD36, and FABP4 Expression and Lipid Accumulation in Renal Proximal Tubular Cells

**DOI:** 10.3390/antiox12091715

**Published:** 2023-09-04

**Authors:** Ke Su, Shui-Ling Zhao, Wen-Xia Yang, Chao-Sheng Lo, Isabelle Chenier, Min-Chun Liao, Yu-Chao Pang, Jun-Zheng Peng, Kana N. Miyata, Jean-Francois Cailhier, Jean Ethier, Jean-Baptiste Lattouf, Janos G. Filep, Julie R. Ingelfinger, Shao-Ling Zhang, John S. D. Chan

**Affiliations:** 1 Centre de Recherche du Centre Hospitalier de l’Université de Montréal (CRCHUM), Département de Médecine, Université de Montréal, 900 Saint Denis Street, Montréal, QC H2X 0A9, Canada; ke.su.chum@ssss.gouv.qc.ca (K.S.); shuiling.zhao@umontreal.ca (S.-L.Z.); wenxia.yang@umontreal.ca (W.-X.Y.); chao-sheng.lo.chum@ssss.gouv.qc.ca (C.-S.L.); isabelle.chenier.chum@ssss.gouv.qc.ca (I.C.); minchun.liao@umontreal.ca (M.-C.L.); yuchao.pang@umontreal.ca (Y.-C.P.); junzheng.peng.chum@ssss.gouv.qc.ca (J.-Z.P.); kana.miyata@health.slu.edu (K.N.M.); jf.cailhier@umontreal.ca (J.-F.C.); jean.ethier.med@ssss.gouv.qc.ca (J.E.); jean-baptiste.lattouf.med@ssss.gouv.qc.ca (J.-B.L.); 2 Centre de Recherche, Hôpital Maisonneuve-Rosemont, Département de Pathologie et Biologie Cellulaire, Université de Montréal, 5415 Boul. de l’Assomption, Montréal, QC H1T 2M4, Canada; janos.g.filep@umontreal.ca; 3 Pediatric Nephrology Unit, Massachusetts General Hospital, Harvard Medical School, 15 Parkman Street, WAC 709, Boston, MA 02114, USA; jingelfinger@nejm.org

**Keywords:** NRF2, SGLT2, CD36

## Abstract

The role(s) of nuclear factor erythroid 2-related factor 2 (NRF2) in diabetic kidney disease (DKD) is/are controversial. We hypothesized that Nrf2 deficiency in type 2 diabetes (T2D) db/db mice (db/db*Nrf2* knockout (KO)) attenuates DKD progression through the down-regulation of angiotensinogen (AGT), sodium-glucose cotransporter-2 (SGLT2), scavenger receptor CD36, and fatty -acid-binding protein 4 (FABP4), and lipid accumulation in renal proximal tubular cells (RPTCs). Db/db*Nrf2* KO mice were studied at 16 weeks of age. Human RPTCs (HK2) with *NRF2* KO via CRISPR-Cas9 genome editing and kidneys from patients with or without T2D were examined. Compared with db/db mice, db/db*Nrf2* KO mice had lower systolic blood pressure, fasting blood glucose, kidney hypertrophy, glomerular filtration rate, urinary albumin/creatinine ratio, tubular lipid droplet accumulation, and decreased expression of AGT, SGLT2, CD36, and FABP4 in RPTCs. Male and female mice had similar results. *NRF2* KO attenuated the stimulatory effect of the Nrf2 activator, oltipraz, on AGT, SGLT2, and CD36 expression and high-glucose/free fatty acid (FFA)-stimulated lipid accumulation in HK2. Kidneys from T2D patients exhibited markedly higher levels of CD36 and FABP4 in RPTCs than kidneys from non-diabetic patients. These data suggest that NRF2 exacerbates DKD through the stimulation of AGT, SGLT2, CD36, and FABP4 expression and lipid accumulation in RPTCs of T2D.

## 1. Introduction

Increased plasma-free fatty acid (FFA) levels and disturbances in lipid metabolism, including increased fatty acid oxidation and accumulation of renal lipid droplets contribute to nephropathy progression in diabetes [1,2,3,4,5]. Thus, understanding the mechanisms underlying lipid accumulation within the kidney is essential in order to prevent the development of diabetic kidney disease (DKD).

CD36, an 88 kiloDalton (kDa) single-chain membrane glycoprotein and a member of the class B scavenger receptor family, has been identified as a long-chain FFA transporter and signal transduction molecule and plays a role in fatty acid oxidation in numerous organs including the kidney [6]. Increased CD36 expression was detected in the kidney cells of patients with diabetes and has been shown to contribute to DKD progression via palmitate-induced tubular apoptosis and tubulo-interstitial fibrosis [3,7,8,9].

FABP4 (fatty-acid-binding protein 4), a small cytoplasmic protein (~14–15 kDa), is a member of the fatty-acid-binding protein family. FABP4 binds with high affinity to long-chain fatty acids and eicosanoids and transports them to cellular components including lipid droplets. Studies have shown a close association between elevated FABP4 and the pathogenesis of metabolic and vascular disease and DKD [10,11].

Sodium-glucose co-transporters (SGLT2 and SGLT1) are secondary active glucose symporters expressed in the apical brush border of the renal proximal tubule (RPT) [12]. In healthy persons, SGLT2 in the S1/S2 segments of RPTs resorb more than 90% of glucose filtered by the glomerulus, whereas SGLT1 is responsible for the resorption of the remaining glucose (10%) in the late RPT (S2/S3 segments) [13]. SGLT2 expression and activity are up-regulated in RPTs of animals with diabetes [14,15] and in proteinuric T2D patients [16,17]. The cardiorenal-protective effects of SGLT2 inhibitors (SGLT2i) have been established in multiple clinical trials in T2D patients [18,19,20,21]. Interestingly, SGLT2i have also been shown to ameliorate FFA-induced lipotoxicity by down-regulating CD36 expression [22]. 

Nuclear factor erythroid 2-related factor 2 (NRF2) is a master regulator of redox balance in cellular cytoprotective responses [23,24]. Studies involving NRF2 activation yielded controversial results in animal models and patients with diabetes [25,26,27,28,29,30,31,32]. We reported that global deletion of *Nrf2* lowered systolic blood pressure (SBP) and decreased angiotensinogen (AGT, the sole precursor of angiotensins) expression in Akita mice, a murine model of T1D [33]. We also showed that Akita *Nrf2*KO mice overexpressing Nrf2 in RPTCs (Akita *Nrf*^−/−^/*Nrf2*^RPTC^ transgenic (Tg) mice) resulted in elevated blood glucose level and increased SGLT2 expression in RPTCs versus Akita *Nrf2*KO mice [17]. 

In the present study, we hypothesized that *Nrf2* deficiency in T2D db/db mice (db/db*Nrf2* KO) would attenuate DKD progression, attenuating hypertension, hyperglycemia, lipid accumulation, and renal morphological changes/dysfunction through the decreased expression of AGT, SGLT2, CD36, and FABP4 in renal proximal tubular cells (RPTCs). We also examined human RPTCs (HK2) with or without *NRF2* KO via CRISPR-Cas9 genome editing, cultured in normal-glucose or high-glucose milieu ± FFA added, and the kidneys of humans with or without diabetes.

## 2. Materials and Methods

### 2.1. Chemicals and Reagents

D-glucose, D-mannitol, and oltipraz (a specific Nrf2 activator) [34] were procured from Sigma-Aldrich Canada Ltd. (Oakville, ON, Canada). Dulbecco’s Modified Eagle’s Medium (DMEM, Catalogue No. 12320) containing normal glucose (NG, 5 mmol/L D-glucose), fetal bovine serum (FBS) and penicillin/streptomycin were procured from Invitrogen, Inc. (Burlington, ON, Canada). HK2 cells (Cat. No. CRL-2190) (an immortalized human RPTC line) were obtained from American Tissue Cell Collection (ATCC) (Manassas, VA, USA) (http://www.atcc.org). The antibodies used are listed in Supplementary Table S1. Oligonucleotides were synthesized by Integrated DNA Technologies, Inc. (Coralville, IA, USA) and are listed in Supplementary Table S2. Restriction and modifying enzymes were procured from commercial sources.

### 2.2. Generation of db/dbNrf2 Knockout (KO) Mice

We cross-bred lean male db/m mice (C57BLKS/J strain) with female homozygous *Nrf2*^−/−^KO mice (B6.129X1-Nfe2l2^tm1Ywk^/J) (Jackson Laboratories, Bar Harbor, ME, USA (http://jaxmice.jax.org) to generate female db/m heterozygous *Nrf2 KO* mice, then back-crossed them with male db/m (C57BLKS/J) for at least 6 generations to obtain db/db homozygous *Nrf2*KO mice (>90% in C57BLKS). (Of note, homozygous *Nrf2* KO mice are viable and fertile, whereas homozygous db/db mice are infertile.) Both sexes of db/db and db/db*Nrf2 KO* mice were studied at age 16 weeks. Age- and sex-matched db/m littermates served as controls. All mice had free access to standard mouse chow and water. Animal care and experimental procedures were approved by the Animal Care Committee of the Centre de Recherche du Centre Hospitalier de l’Université de Montréal (CRCHUM) and followed the National Institutes of Health (NIH)’s Principles of Laboratory Animal Care (Publication No. 85–23, revised 1985: http://grants1.nih.gov/grants/olaw/references/phspol.htm, accessed on 29 June 2023).

### 2.3. Pathophysiology 

Systolic blood pressure (SBP) was measured via a BP-2000 tail-cuff pressure monitor (Visitech Systems, Apex, NC, USA) at least 2 to 3 times per week [17,33]. Each mouse was accustomed to the procedure for at least 15 to 20 min per day for 5 days before the first SBP measurement at the age of 16 weeks. SBP values are presented as means ± SEM of 2–3 determinations/mouse/group.

Fasting blood glucose (FBG) levels were measured in mice with the Accu-Chek Performa System (Roche Diagnostics, Laval, QC, Canada) after fasting 4 to 6 h or with a glucose colorimetric detection kit (Cayman Chemical, Ann Habor, MI, USA) at the age of 16 weeks, as described previously [17,33].

Lean body mass (muscle), fat mass, and total body water were determined using EchoMRI-100 body composition analyzer (EchoMRI, Houston, TX, USA).

Glomerular filtration rate (GFR) was estimated with fluorescein isothiocyanate (FITC) inulin, as recommended by the Animal Models of Diabetic Complications Consortium (http://www.diacomp.org/), as described previously [17,33,35].

The mice were housed individually in metabolic cages for 6 h during the daytime for urine collection prior to euthanasia at 16 weeks. Urine samples were assayed for albumin and creatinine via albumin enzyme-linked immunosorbent assay (ELISA, Albuwell and Creatinine Companion, Exocell, Inc., Philadelphia, PA, USA) [17,33], respectively.

Following euthanasia, the kidneys were removed, decapsulated, and weighed. Left kidneys were used for histology and immunostaining and the right kidneys were processed immediately for isolation of renal proximal tubules (RPTs) via Percoll gradient [17,33,36]. Aliquots of freshly isolated RPTs were assessed for total RNA and protein.

### 2.4. Histology 

A total of 4–5 sections per kidney and 6 mouse kidneys per group were immunostained using the standard avidin-biotin-peroxidase complex method (ABC Staining, Santa Cruz Biotechnology, Santa Cruz, CA, USA) [17,33]. Kidney sections were counterstained with hematoxylin and then analyzed via light microscopy by 2 investigators blinded to treatment groups.

Periodic acid–Schiff (PAS) staining was also performed to assess kidney morphology. Histological changes including glomerular tuft volume, RPTC volume, and tubular luminal area were determined via the methods of Weibel and Gundersen [37,38] by using an image analysis software (Motics Images Plus 2.0; Motic, Richmond, BC, Canada). Tubular injury score was determined as described by Chen et al. [39].

RPTs were assessed for oxidative stress by staining with 8-hydroxyguanosine (8-OHdG) and dihydroethidium (DHE) (Sigma), whereas lipid droplets were assessed via Oil Red O (Oil Red O staining kit, ab150678, Abcam, Toronto, ON, Canada) of frozen kidney sections, respectively. Semi-quantification of the relative staining was performed by using Image J software Version 1.53K (https://rsb.info.nih.gov/ij, accessed on 29 June 2023).

Immunofluorescence (IF) staining for SGLT2 and FABP4 was performed on 4-μm tissue sections from mouse kidneys fixed in formalin and embedded in paraffin followed by staining with ALEXA FLUOR-594-labeled secondary antibody (Invitrogen). Proximal tubules were identified via fluorescein-labeled lotus tetragonolobus lectin (LTL, a marker of RPT [40]) (Vector Labs, Burlingame, CA, USA). Image quantification and merging were assessed via Image J software (http://rsb.info.nih.gov/ij/). The pixel intensity of SGLT2 or FABP4 was divided by LTL intensity to quantify the amount of SGLT2 or FABP4 expression. Six mice per group were analyzed to calculate the average ratio.

### 2.5. Western Blotting

Western blotting (WB) was performed as described previously [17,33]. ImageQuant software (version 5.1, Molecular Dynamics, Sunnyvale, CA, USA) was used to quantify the relative densities of NRF2, KEAP 1 (Kelch-like, ECH-associated protein 1), HO-1 (heme oxygenase-1), AGT, SGLT2, CD36, and β-actin bands.

### 2.6. Real-Time Quantitative Polymerase Chain Reaction (RT-qPCR)

*Nrf2, Keap1, HO-1, Nox4* (NADPH Oxidase 4), *Cat* (Catalase), *Agt, Sglt2, CD36, Fabp4*, and *Rpl 13a* (ribosomal protein L13a) mRNA levels in RPTs were quantified via RT-qPCR with specific primers (Supplementary Table S2) [17,33].

### 2.7. Cell Culture

HK2 cells were cultured as described previously [17,33,41].

*NRF2* KO in HK2 was performed via the CRISPR-Cas9 genome editing method provided by Invitrogen (TrueGuide™) as previously described [42].

To test the effect of genetic deletion of *NRF2* on AGT, SGLT2, and CD36 expression in HK2 and HK2 ± NRF2 KO were harvested after 24 h of culture in serum-free DMEM (5 mM D-glucose plus 30 mM D-mannitol) ± 45 µM oltipraz or in 35 mM D-glucose DMEM ± 200 µM palmitate/oleate-BSA [41,42]. The palmitate/oleate-BSA was prepared as described by Roche E. et al. [43].

WB and RT-qPCR were used to quantify expression of AGT, SGLT2, and CD36 protein and mRNA, respectively. Oil Red O staining was used to assess lipid droplets in HK2 ± NRF2 KO cultured in normal or high glucose ± 200 µM palmitate/oleate-BSA added, respectively.

### 2.8. Immunostaining of CD36 and FABP4 on Human Kidney Specimens

Nephrectomy specimens (paraffin sections) from patients with or without diabetes obtained from the Department of Pathology, CHUM were immunostained for CD36 and FABP4. The CHUM Clinical Research Ethics Committee approved the study. All patients had undergone nephrectomy for kidney cancer and gave signed written informed consent for the use of their kidney tissue for research. Patients’ clinical characteristics have been published previously [17] (Figure S4).

### 2.9. Statistical Analysis

The data are expressed as means ±SEM. Statistical comparisons were made via the Student’s *t*-test or 1-way analysis of variance and the Bonferroni correction as appropriate. *p* < 0.05 values were considered to be statistically significant.

## 3. Results

### 3.1. NRF2 Expression in db/db Nrf2KO Mouse Ear and Kidney Tissue

**Figure 1:**
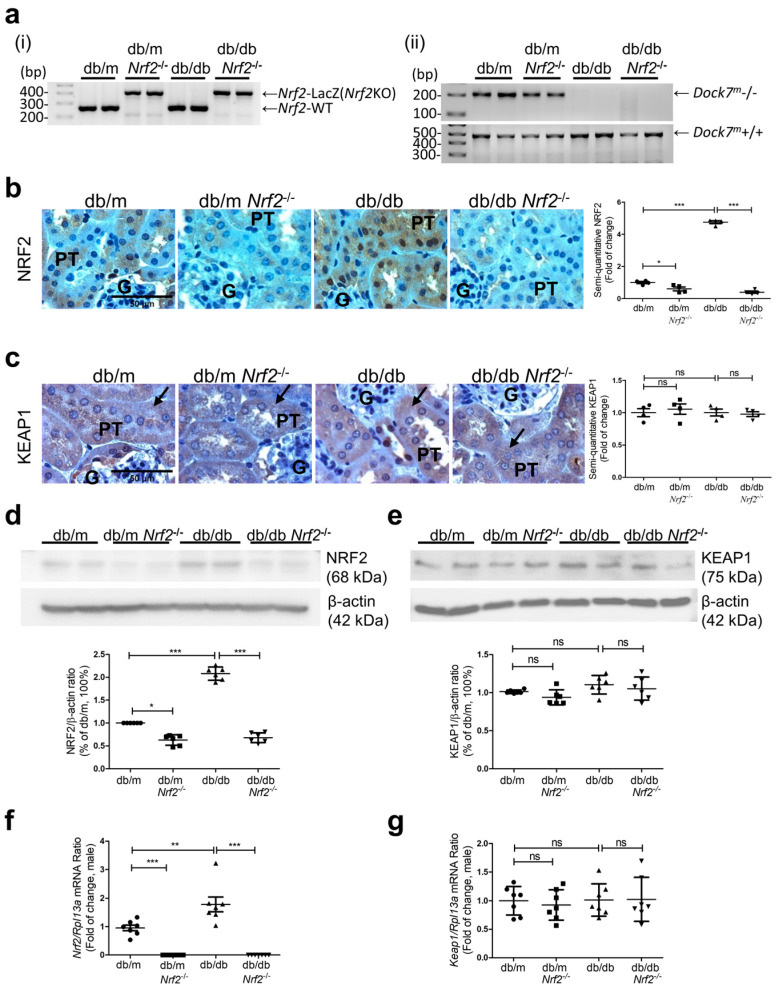
Generation of db/db*Nrf2* KO Mice. (**a**) Genotyping of (panel (**i**)) *Nrf2-LacZ* (*Nrf2* KO) and wild-type *Nrf2* gene in male db/m, db/m*Nrf2* KO, db/db, and db/db*Nrf2* KO mice and (panel (**ii**)) db/m mice identified by the presence of a heterozygous Dock7 gene, whereas db/db mice identified by the presence of a homozygous Dock7 gene. Immunohistochemical staining for NRF2 (**b**) and KEAP1 (**c**) expression in kidney sections (arrows indicate proximal tubules; magnification ×600), WB analysis of NRF2 (**d**) and KEAP1 (**e**) protein expression, and RT-qPCR analysis of *Nrf2* (**f**) and *Keap1* (**g**) mRNA levels in RPTs of male db/m, db/m*Nrf2* KO, db/db, and db/db*Nrf2* KO mice at age 16 weeks. P: proximal tubule, G: glomerulus. IHC (*n* = 4 per group) and WB (*n* = 6 per group); mRNA expression (*n* = 7 per group). Statistics were measured via one-way ANOVA followed by Bonferroni post hoc test. * *p* < 0.05; ** *p* < 0.01; *** *p* < 0.005; ns, not significant.

PCR analysis confirmed the presence of *Nrf2* gene in the ear tissue of db/m and db/db mice but not in db/m*Nrf2*^−/−^(KO) and db/db*Nrf2* KO mice (Figure 1a (panel i)). Db/db mice are identified by the presence of the Dock7 gene, which was introgressed into a mutant leptin receptor (Figure 1a (panel ii)). Immunostaining for NRF2 was more pronounced in nuclei of RPTCs from db/db mice than in db/m mice and was not detectable in db/m*Nrf2* KO and db/db*Nrf2* KO mice of both sexes (Figure 1b and Supplementary Figure S1a). In contrast, KEAP1 immunostaining results were similar in the groups studied (Figure 1c). NRF2 and KEAP1 expression in RPTs assessed via respective WB (Figure 1d,e and Supplementary Figure S1b) and RT-qPCR of *Nrf2* and *Keap1* (Figure 1f,g and Supplementary Figure S1c) were consistent with these findings.

### 3.2. Physiological Measurements in Mice

**Figure 2:**
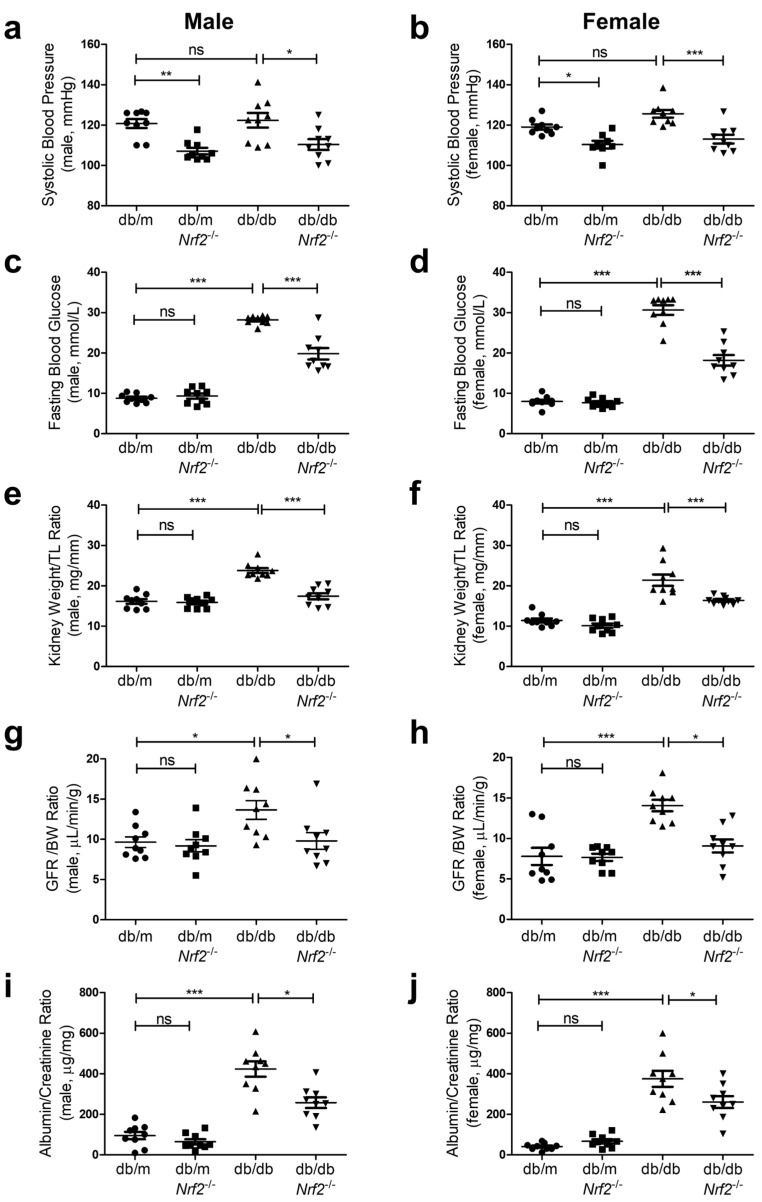
Effects of *Nrf2* KO on physiological parameters of db/db Mice (male and female) at 16 weeks of age. (**a**,**b**) Systolic blood pressure (SBP); (**c**,**d**) fasting blood glucose (FBG); (**e**,**f**) KW/tibial length (TL) ratio; (**g**,**h**) glomerular filtration rate (GFR); and (**i**,**j**) albumin–creatinine ratio (ACR) from db/m, db/mNrf2 KO, db/db, and db/dbNrf2 KO mice. Values are mean + SEM; n = 9 per group. Statistics were obtained via one-way ANOVA followed by Bonferroni post hoc test. * *p* < 0.05; ** *p* < 0.01; *** *p* < 0.005; ns, not significant.

Genetic deletion of *Nrf2* significantly decreased SBP (on average, SBP was ~10 mm Hg lower) in db/m*Nrf2* KO and db/db*Nrf2* KO mice than respective db/m and db/db mice at 16 weeks in both sexes (Figure 2a,b). However, SBP did not differ significantly in db/m and db/db mice despite slight increases in db/db as compared to db/m mice.

FBG levels were significantly higher in db/db than in db/m mice in both sexes (Figure 2c,d). Deletion of *Nrf2* resulted in significantly lower FBG levels in db/db*Nrf2* KO than in db/db mice, whereas FBG levels were similar in both male and female db/m and db/m*Nrf2* KO mice (Figure 2c,d).

Both male and female db/db and db/db*Nrf2* KO mice had significantly higher body weights (BWs) than respective db/m and db/m*Nrf2* KO mice (Supplementary Figure S2a and Figure 2b). While *Nrf2* KO had no detectable effects on BW in db/db mice, it decreased the kidney weight (KW)/tibial length (TL) ratio (Figure 2e,f), glomerular filtration rate (GFR)/BW ratio (Figure 2g,h), and urinary albumin–creatinine ratio (ACR) (Figure 2i,j) in db/db*Nrf2* KO vs. db/db mice of both sexes. These parameters did not differ between db/m and db/m*Nrf2* KO mice (Figure 2e–j).

Fat content/BW (Supplementary Figure S2c and Figure 2d), lean mass/BW (Supplementary Figure S2e and Figure 2f), and total water content/BW (Supplementary Figure S2g and Figure 2h) were similar in db/m and db/m*Nrf2* KO mice, whereas significant increases in fat content/BW and decreases in lean mass/BW and total water content/BW were detected in db/db and db/db*Nrf2* KO mice vs. respective db/m and db/m*Nrf2* KO mice of both sexes.

### 3.3. Histology and Immunostaining

**Figure 3:**
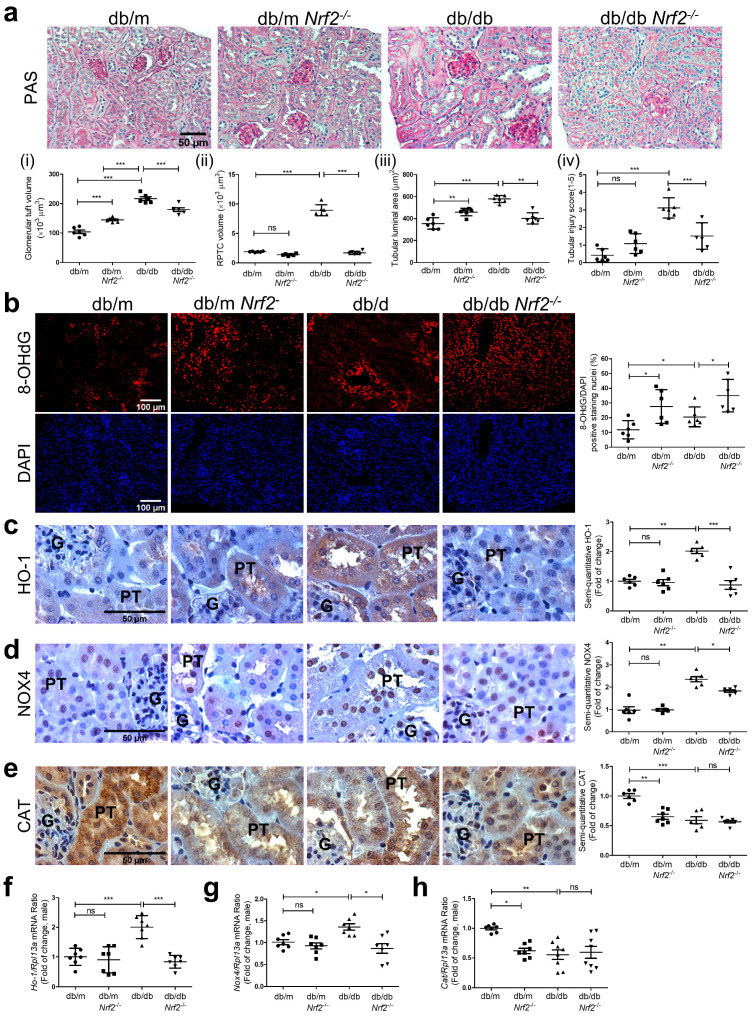
Kidney morphology and oxidative stress in male db/db mouse kidneys at 16 weeks of age. (**a**) PAS staining (×200) and semi-quantitation of glomerular tuft volume (**i**), RPTC volume (**ii**), tubular luminal area (**iii**), and tubular injury score (**iv**); (**b**) 8-OHdG staining (×100) and semi-quantitation; (**c**) HO-1 immunostaining (×600) and semi-quantitation; (**d**) NOX-4 immunostaining (×600) and semi-quantitation; (**e**) Catalase (CAT) immunostaining (×600) and semi-quantitation in kidney sections from male db/m, db/m*Nrf2* KO, db/db, and db/db*Nrf2* KO mouse kidneys at 16 weeks of age. RT-qPCR of HO-1 (**f**), Nox4 (**g**), and Cat (**h**) expression in RPTs of db/m, db/m*Nrf2* KO, db/db, and db/db*Nrf2* KO mice. Values are expressed as mean ± SEM; n = 6 per group for staining. n = 7–8 per group for mRNA expression. Statistics were measured via one-way ANOVA followed by Bonferroni post hoc test. * *p* < 0.05; ** *p* < 0.01; *** *p* < 0.005 vs. db/m; ns, not significant.

The kidneys of db/db mice exhibited structural damage vs. db/m mice (Figure 3a), which is consistent with our earlier observations [44]. Histological changes including enlarged glomerular tuft volume, tubular luminal area, and RPTC volume in db/db mice, were attenuated in db/db*Nrf2* KO mice (Figure 3(ai,aii,aiii), respectively). Tubular injury scores confirmed these observations (Figure 3(aiv)).

Significantly stronger staining for 8-OHdG (Figure 3b) and DHE (Supplementary Figure S3) was detected in RPTs of db/db vs. db/m and db/m*Nrf2* KO mice, but it did not differ from that in db/db*Nrf2* KO mice. In contrast, immunostaining for HO-1 (Figure 3c) and NOX-4 (Figure 3d) in RPTs were significantly increased in RPTs from db/db mice compared to db/m and db/m*Nrf2* KO mice, whereas these changes were attenuated in db/db*Nrf2* KO mice. In contrast, CAT immunostaining was significantly higher in RPTs of db/m than in db/m*Nrf2* KO, db/db, or db/db*Nrf2* KO mice (Figure 3e). These findings were confirmed via semi-quantification of 8-OHdG (Figure 3b) and DHE (Supplementary Figure S3) staining and immunostaining for HO-1 (Figure 3c), NOX-4 (Figure 3d), and CAT (Figure 3e) as well as via RT-qPCR for *HO-1* (Figure 3f), *Nox-4* (Figure 3g), and *Cat* (Figure 3h) from isolated mouse RPTs.

### 3.4. Nrf2 Deletion on AGT and SGLT2 Expression in db/db Mice

**Figure 4:**
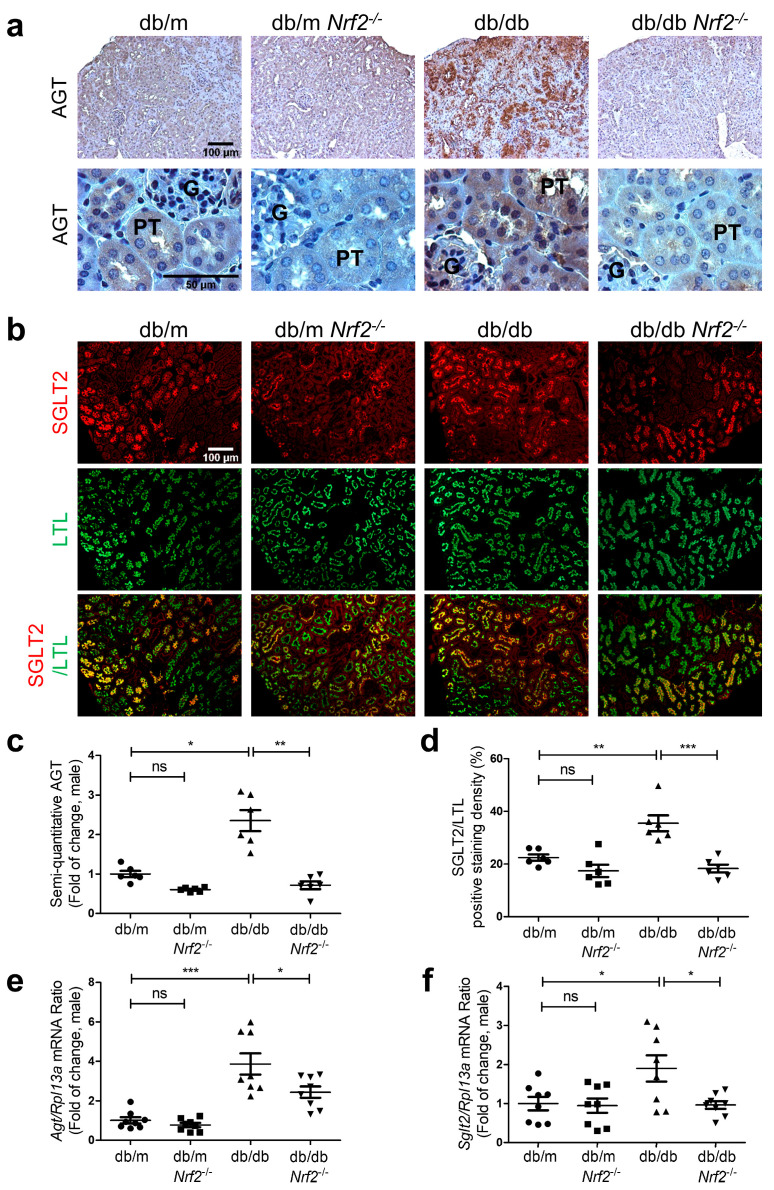
AGT and SGLT2 expression in male db/db mouse kidneys at week 16 immunohistochemical staining of AGT (**a**), immunofluorescent staining of SGLT2 and LTL (**b**), semi-quantification of AGT immunostaining (**c**), and SGLT2/LTL ratio (**d**) in mouse kidneys. RT-qPCR analysis of *Agt* (**e**) and *Sglt2* (**f**) levels in RPTs of db/m, db/m*Nrf2* KO, db/db, and db/db*Nrf2* KO mice. Values are expressed as mean ± SEM; n = 6 per group for staining; n = 8 per group for mRNA expression. Statistics were obtained via one-way ANOVA followed by Bonferroni post hoc test. * *p* < 0.05; ** *p* < 0.01; *** *p* < 0.005 vs. db/m; ns, not significant.

AGT immunostaining in RPTs of db/db mice was more pronounced vs. db/db*Nrf2* KO mice (Figure 4a and Supplementary Figure S1d). Co-immunofluorescence (IF) staining for SGLT2 and LTL revealed higher SGLT2/LTL ratios in RPTs from db/db vs. db/m mice of both sexes (Figure 4b and Supplementary Figure S1e, respectively). Db/db*Nrf2* KO mice exhibited lower SGLT2/LTL ratios than db/db mice. No significant changes were detected between db/m and db/m*Nrf2* KO mouse kidneys. Semi-quantitation of immunostaining images of AGT (Figure 4c) and SGLT2/LTL (Figure 4d and Supplementary Figure S1f) as well as RT-qPCR of their respective mRNAs in isolated RPTs (Figure 4e,f and Supplementary Figure S1g,h) confirmed these observations.

### 3.5. Nrf2 Deficiency on Lipid Accumulation and Expression of CD36 and FABP4 in db/db Mice

**Figure 5:**
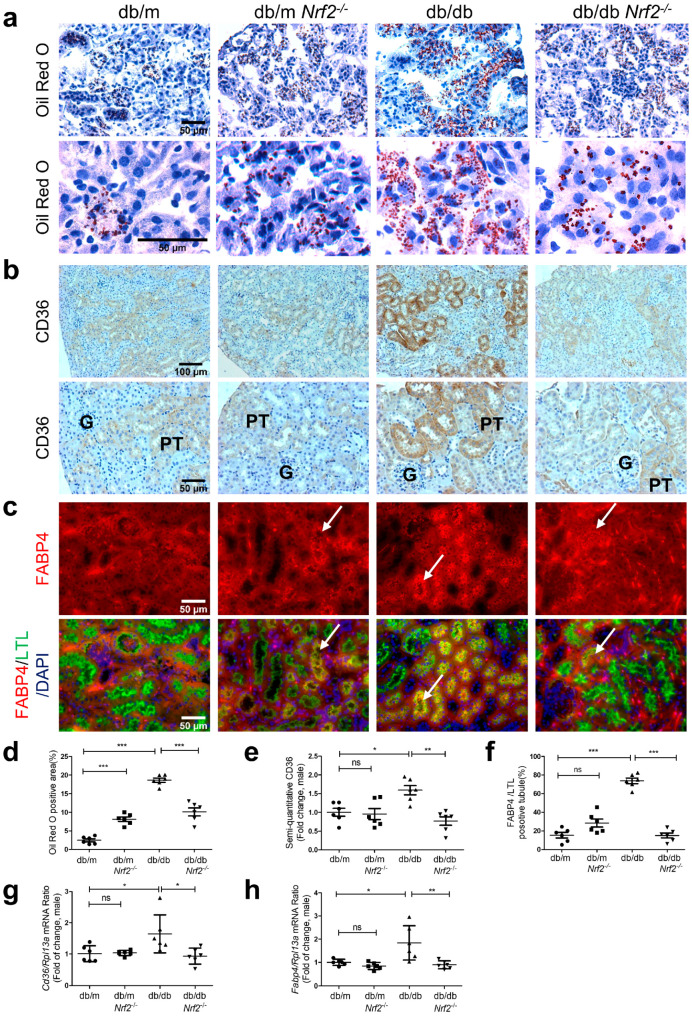
Oil Red O Staining and expression of CD36 and FABP4 in male db/db mouse kidneys at week 16. (**a**) Oil Red O staining (×200, ×600); (**b**) CD36 immunostaining (×100, ×200); (**c**) FABP4/LTL immunofluorescence staining (×200) (white arrows indicate proximal tubules); (**d**) semi-quantification of Oil Red O staining, CD36 immunostaining (**e**); and immunofluorescent staining of FABP4/LTL ratio (**f**) in mouse kidneys. RT-qPCR analysis of *CD36* (**g**) and *Fabp4* (**h**) levels in RPTs of db/m, db/m*Nrf2* KO, db/db, and db/db*Nrf2* KO mice. Values are expressed as mean ± SEM; n = 6 per group for staining; n = 6 per group for mRNA expression. Statistics were obtained via one-way ANOVA followed by Bonferroni post hoc test. * *p* < 0.05; ** *p* < 0.01; *** *p* < 0.005 vs. db/m; ns, not significant.

Oil Red O staining increased in renal tubules of db/db vs. db/m mice (Figure 5a), and this was markedly decreased in the renal tubules of db/db*Nrf2* KO mice. Immunostaining of CD36 revealed higher expression in RPTs from db/db vs. db/m and db/m*Nrf2*KO mice (Figure 5b), whereas db/db*Nrf2*KO mice exhibited lower CD36 expression than db/db mice. Similar changes were detected for FABP4 immunostaining, with significant increases in db/db vs. db/m mice (Figure 5c), whereas FABP4 immunostaining was lower in db/db*Nrf2*KO mice than in db/db mice. Semi-quantitation of Oil Red O staining (Figure 5d), CD36 (Figure 5e), and FABP4 (Figure 5f) immunostaining, and RT-qPCR of *CD36* (Figure 5g) and *Fabp4* (Figure 5h) confirmed these observations.

### 3.6. Effects of NRF2 KO on AGT, SGLT2, and CD36 Expression in HK2

**Figure 6:**
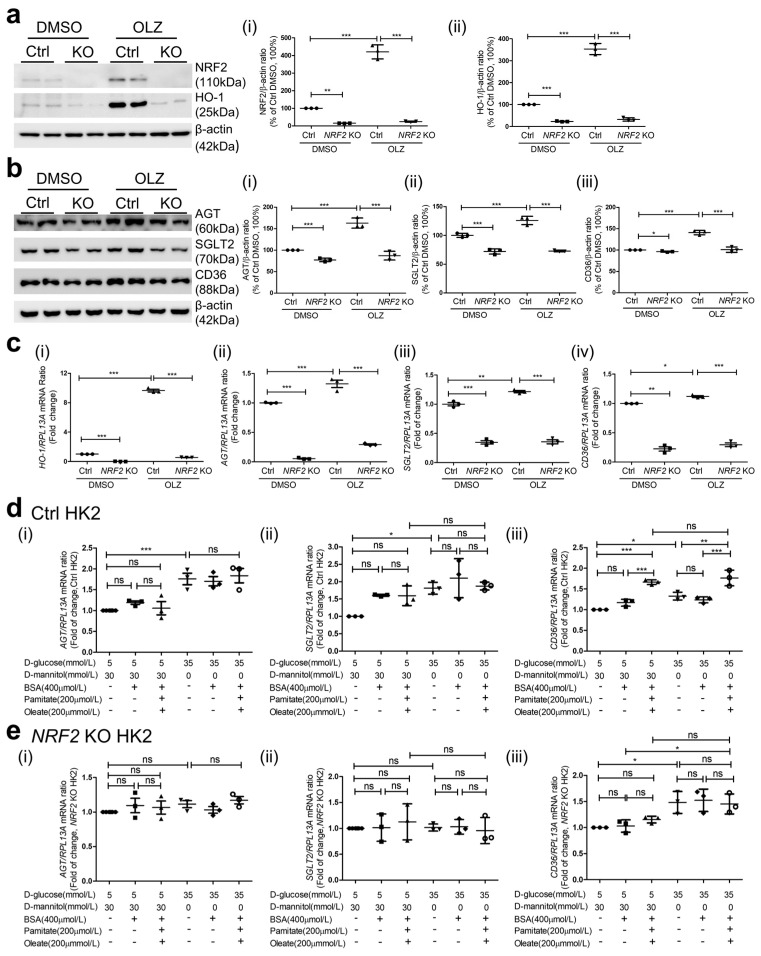
*AGT, SGLT2*, and *CD36* expression in HK2 with or without *NRF2* KO WB and semi-quantitation of NRF2 and HO-1 (**a**) (**i**) NRF2; (**ii**) HO-1 and (**b**) (**i**) AGT; (**ii**) SGLT2 and (**iii**) CD36 in different clones of HK2 with or without *NRF2* KO via CRISPR gRNA in the presence or absence of 45 µM oltipraz (OLZ). (**c**) RT-qPCR of *HO-1* (**i**), *AGT* (**ii**), *SGLT2* (**iii**), and *CD36* (**iv**) in HK2 with or without *NRF2*KO cultured ± Olz added. (**d**) RT-qPCR of *AGT*, *SGLT2*, and *CD36* in HK2 without *NRF2* KO ((**i**), (**ii**), and (**iii**), respectively), and (**e**) RT-qPCR of *AGT*, *SGLT2*, and *CD36* in HK2 with *NRF2* KO ((**i**), (**ii**), and (**iii**), respectively) cultured in normal glucose (NG, 5 mM D-glucose + 30 mM D-mannitol) or high glucose (HG, 35 mM D-glucose) ± palmitate/oleate (200 µM). Experiments were repeated at least 3 times in duplicates. Values are mean ± SEM; n = 3. Statistics were obtained via one-way ANOVA followed by Bonferroni post hoc test. * *p* < 0.05, ** *p* < 0.01, *** *p* < 0.005; ns, not significant. HK2*NRF2* KO versus HK2 control (Ctrl). Note: Student *t*-test was used to analyze HK2*NRF2* KO versus Ctrl with the treatment of DMSO in (**b**(**i**,**iii**)). * *p* < 0.05; *** *p* < 0.005.

NRF2 and HO-1 expression in HK2 with *NRF2* KO was undetectable via immunoblotting (Figure 6(ai,aii), respectively). These cells also exhibited lower AGT, SGLT2, and CD36 expression (Figure 6(bi,bii,biii), respectively) vs. HK2 cultured in normal glucose (NG, 5 mM D-glucose) DMEM. As anticipated, oltipraz stimulated the expression of *HO-1, AGT*, *SGLT2*, and *CD36* expression in HK2, whereas it failed to induce *HO-1*, *AGT*, *SGLT2*, *CD36* expression in HK2 with *NRF2* KO (Figure 6(ci,cii,ciii,civ), respectively). Culture in high glucose (HG, 35 mM D-glucose) significantly increased the expression of *AGT*, *SGLT2*, and *CD36* expression vs. HK2 cultured in NG (Figure 6(di,dii,diii), respectively). However, FFA (200 µM palmitate/oleate-BSA) failed to stimulate *AGT* and *SGLT2* expression (Figure 6(di,dii)) but stimulated *CD36* expression in both NG and HG media, and its stimulatory effect was enhanced in HG in HK2 (Figure 6(diii)). This stimulatory effect of FFA on *AGT*, *SGLT2*, and *CD36* expression was prevented in HK2 with *NRF2* KO (Figure 6(ei,eii,eiii), respectively). Intriguingly, HG stimulated *CD36* expression in *NRF2* KO HK2 in both NG and HG (Figure 6(eiii)).

### 3.7. Oil Red O Staining in HK-2 with or without NRF2 KO

**Figure 7:**
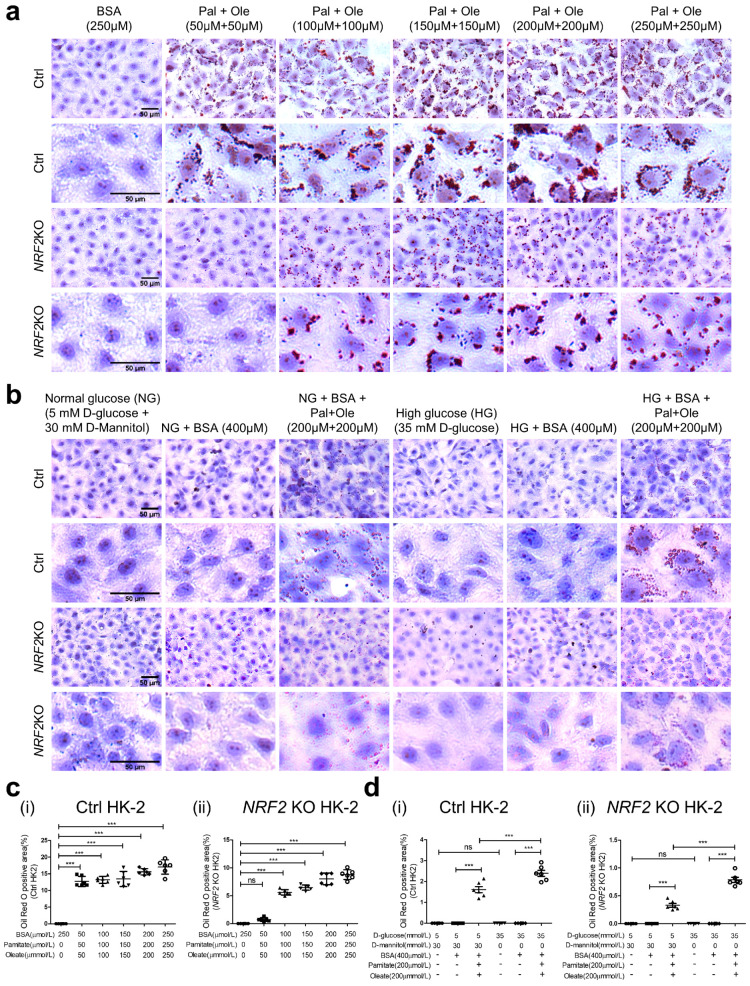
Oil Red O Staining in HK-2 with or without *NRF2* KO. (**a**) Oil Red O staining in HK2 with or without *NRF2* KO cultured in NG in FFA-depleted BSA or different concentrations of FFA (palmitate/oleate). (**b**) Oil Red O staining in HK2 with or without *NRF2* KO cultured in NG or HG ± FFA-depleted BSA (400 µM) or palmitate (200 µM)/oleate (200 µM). (**c**) and (**d**) Semi-quantification of Oil Red O staining in (i) HK2 control or (ii) HK2 with *NRF2* KO cultured in NG or HG ± FFA-depleted BSA or palmitate/oleate. Values are mean ± SEM; n = 3. Statistics were obtained via one-way ANOVA followed by Bonferroni post hoc test. *** *p* < 0.005; ns, not significant. HK2*NRF2* KO versus HK2 control (Ctrl).

While BSA did not increase oil droplet accumulation, FFA enhanced the accumulation of oil droplets in HK2 cells in NG in a dose-dependent manner, which was attenuated in *NRF2* KO HK2 cells (Figure 7a). Moreover, HG did not increase oil droplet accumulation but FFA enhanced accumulation of oil droplets in HK2 cells in NG and HG, which was attenuated in *NRF2* KO HK2 cells (Figure 7b) and confirmed by the quantitation of oil droplets accumulation (Figure 7(di,dii)).

### 3.8. CD36 and FABP4 Immunostaining in Human Kidney Sections

**Figure 8:**
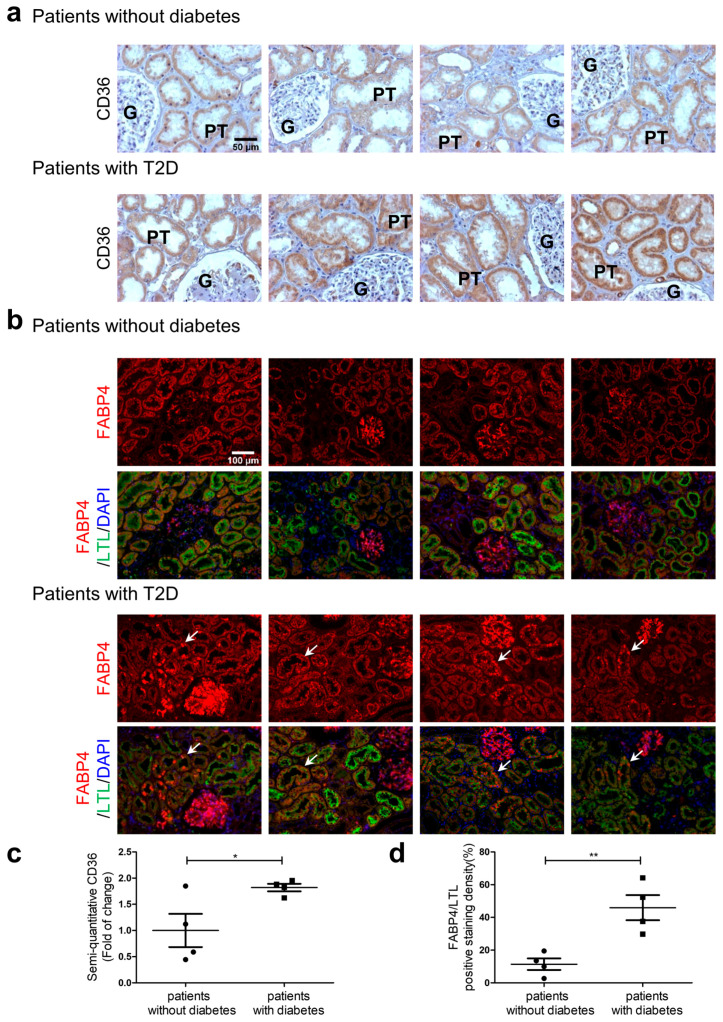
CD36 and FABP4 expression in human kidney sections**.** Samples were obtained from areas without tumors from patients who underwent nephrectomy for carcinoma of the kidney. CD36 immunostaining (**a**) and FABP4/LTL/DAPI immunofluorescent staining (**b**) from 4 patients without diabetes and from 4 patients with diabetes (white arrows indicate proximal tubules). (**c**) Semi-quantitation of CD36 and (**d**) FABP4/LTL/DAPI staining from patients ± diabetes. CD36 magnification ×200 and FABP4 magnification ×100. G, glomerulus; and P, proximal tubule. * *p* < 0.05; ** *p* < 0.01 vs. Patients without diabetes (Student’s unpaired *t*-test).

Increased CD36 and FABP4 immunostaining were detected in kidney specimens from diabetic patients vs. those without diabetes (Figure 8a and Figure 8b, respectively) and confirmed via quantitation of immunostaining (Figure 8c and Figure 8d), respectively). These observations are consistent with CD36 and FABP4 changes observed in the RPTs of db/db mice.

## 4. Discussion

Our results document that the genetic deletion of *Nrf2* in db/db mice was associated with attenuated DKD features (SBP, FBG, GFR, urinary ACR, tubular lipid accumulation, kidney injury) in tandem with decreased expression of AGT, SGLT2, and CD36 protein and mRNA in RPTCs. HK2 cells with *NRF2* KO also exhibited lower expression of AGT, SGLT2, and CD36 and did not respond to the stimulatory effect of oltipraz or FFA on the expression of these proteins/genes. These data would appear to link NRF2 activation, intrarenal RAS and SGLT2 expression and tubular lipid accumulation, to the exacerbation of hypertension, dysglycemia, and kidney injury in T2D.

The db/db mouse (LepR^db/db^in BKS background) is an established T2D murine model [45] (http://www.diacomp.org/). We found elevated NRF2 and HO-1 expression in RPTs of 16-week-old db/db mice, with no detectable differences in KEAP1 expression. In contrast, HO-1 expression was decreased in the RPTs of db/m*Nrf2* KO and db/db*Nrf2* KO mice vs. respective db/m and db/db mice. These data confirmed that HO-1 is a downstream target of NRF2 and confirmed the usefulness of db/db*Nrf2* KO mice as a preclinical model to study the role of NRF2 in T2D.

Significantly lower SBP was found in both male and female db/m*Nrf2* KO and db/db*Nrf2* KO mice vs. respective db/m and db/db mice. Although SBP was slightly higher in db/db mice vs. db/m mice, it did not reach statistical significance. These might be due to the short duration (15–20 min per day) of BP measurement via the tail-cuff pressure monitor. More sensitive BP measurements such as telemetry for continuous 24–48 h monitoring are required to detect the differences in BP between these mice.

Significantly lower FBG levels, KW/TL, urinary ACR, and GFR were found in db/db*Nrf2* KO mice vs. db/db mice of both sexes. However, these parameters were similar in db/m mice and db/m*Nrf2* KO mice at 16 weeks of age, implying that *Nrf2* deficiency may improve these parameters in db/db mice via lowering blood glucose. At present, the exact reason for the decline in the GFR following *Nrf2* deletion is not clear. However, since *Nrf2* deletion down-regulated SGLT2 and AGT/Ang II expression in the RPTs, we speculate that the reduction in GFR and SBP could be attributed, at least in part, to lowering FBG levels and SGLT2 expression in RPTs. This would lead to vasoconstriction in the afferent arteriole induced by the restoration of the tubulo-glomerular feedback (TGF) [46,47] and the down-regulation of intrarenal AGT/Ang II expression, causing vasodilation in the efferent arteriole to lower intraglomerular pressure [48,49]. Cumulatively, all these factors could lead to decreases in GFR, SBP, and urinary ACR. No significant changes of fat mass/BW, lean mass/BW, and total water content/BW were observed between db/db*Nrf2* KO and db/db mice as well as between db/m and db/m*Nrf2* KO mice. These findings lend further support to the idea that the beneficial effects of *Nrf2* deficiency on BG level might be predominately due to the loss of NRF2 action in the kidney rather than in fat or muscle tissue. It remains to be determined whether *Nrf2* deletion in db/db mice could affect insulin sensitivity or production, food consumption, circulating levels of triglycerides, and fatty acids. Works are ongoing to address these issues.

Increased 8-OHdG staining and DHE (markers of oxidative stress) were found in RPTs of db/db mice vs. db/m, but the staining was lower than in db/m*Nrf2* KO mice and db/db*Nrf2* KO mice. Increased NOX4 expression was also observed in db/db mice vs. db/m and db/m*Nrf2* KO mice and attenuated in db/db*Nrf2* KO mice. We do not presently understand why *Nrf2* deletion exhibited higher oxidative stress in RPTs of db/m*Nrf2* KO and db/db*Nrf2* KO mice vs. db/db mice. We have observed, however, that *Cat* mRNA expression was lower in RPTs of db/db vs. db/m mice, and deletion of *Nrf2* did not change *Cat* expression in db/db*Nrf2* KO mice vs. db/db mice. These data indicate that hyperglycemia/hyperlipidemia and *Nrf2* deletion would alter relative expression and activity of Nox4 and Cat, thereby enhancing oxidative stress and likely contributing to renal injury in db/db mice.

The mechanism(s) by which *Nrf2* deficiency leads to the downregulation of renal AGT expression in diabetes remain(s) undefined. By employing gene deletion analysis and site-directed mutagenesis, we have shown that NRF2 binds to two NRF2-*responsive elements (REs)* in the 5′-flanking region of the *Agt* promoter and that deletion of both NRF2-*RE*s is more effective in inhibiting the stimulatory effect of oltipraz than deletion of either NRF2-*RE* alone [33,50]. These findings would indicate that NRF2 regulates *Agt* gene expression at the transcriptional level.

Our present results demonstrate that *Nrf2* deficiency lowers SGLT2 expression in db/db*Nrf2* KO vs. db/db mice. These findings are consistent with our previous studies in that genetic deletion of *Nrf2* decreases the expression of SGLT2 in Akita*Nrf2* KO mice and NRF2 stimulates *Sglt2* transcription via the NRF2-*RE* in the *Sglt2* gene promoter, as shown by gene deletion, site-directed mutagenesis, gel mobility shift, and chromatin-immunoprecipitation (ChIP) assays [17], further supporting the pathophysiological role of NRF2-mediated up-regulation of *Sglt2* expression in diabetic mice.

Studies on the protective role of NRF2 in renal diseases yielded conflicting results [51]. In contrast to our findings that *Nrf2* deficiency improves kidney injury in db/db mice, Jiang et al. [30] and Yok et al. [52] reported that streptozotocin (STZ)-induced diabetic *Nrf2*^−/−^ mice displayed increased glomerular fibrosis and lower creatinine clearance vs. non-diabetic mice. More recently, Liu et al. [53] reported that *Nrf2* deficiency led to deterioration of DKD with no change of SGLT2 expression in Akita*Nrf^−^*^/*−*^ mice vs. Akita mice. The authors also found that blood glucose levels were elevated and fractional excretion of glucose was lower in Akita *Nrf2*^−/−^ mice vs. Akita mice, indicating that SGLT2 expression is not under NRF2 regulation. At present, we do not have a clear explanation for these discrepancies. However, Liu et al. [53] utilized a unique *Nrf2* KO *Nfe212*^tm1Mym^ mouse line established by their laboratory [54], whereas we used *Nrf2*^−/−^ KO mice (B6.129X1-Nfe2l2^tm1Ywk^/J) procured from The Jackson Laboratory (http://jaxmice.jax.org). Thus, it remains to be investigated whether differences in mutation of *Nrf2* gene in these *Nrf2*^−/−^ mice could explain these discrepancies.

Consistently, our in vitro results in HK2 with *NRF2* KO confirm Nrf2 regulation of endogenous AGT, SGLT2, and CD36 expression and that *NRF2* deletion renders HK2 unresponsive to oltipraz stimulation of AGT, SGLT2, and CD36 protein and mRNA expression. Intriguingly, we found that HG could stimulate *CD36* expression but not *AGT* and *SGLT2* expression in HK2 with *NRF2* KO. These results suggest that additional signaling pathway(s) might mediate HG stimulation of *CD36* expression which is independent of NRF2/KEAP1 signaling. This possibility is supported by the recent study of Huang et al. [22] that SGLT2i ameliorates FFA-induced lipotoxicity in RPTCs via the peroxisome proliferator-activated receptor-gamma (PPARγ)/CD36 pathway in obese mice, suggesting that SGLT2 up-regulation by HG may stimulate CD36 expression via PPARγ.

Our data revealed that db/db*Nrf2* KO mice exhibit decreased tubular lipid droplets and CD36 and FABP4 expression vs. db/db mice, implying a role for NRF2 in the regulation of tubular lipid accumulation via increased CD36 and FABP4 expression. However, the mechanisms of CD36 and FABP4 regulation in RPTCs remain to be defined. Studies by Maruyama et al. [55] show that NRF2 stimulates *CD36* gene transcription in macrophages, and Sussan et al. [56] reported decreased CD36 expression in macrophages and in atherosclerotic plaques from Apol^−/−^ Nrf2^−/−^ mice, indicating that NRF2 regulates *CD36* expression at the transcriptional level. We have found that Fabp4 protein and mRNA expression is also down-regulated in the RPTs of db/db*Nrf2* KO mice. It remains to be investigated whether NRF2 regulates *Fabp4* expression at the transcriptional level. Our results may help explain the potentially harmful effects of NRF2 activation (with bardoxolone methyl (bardoxolone)) reported in T2D patients with advanced CKD (BEACON trial) [29,32]. The BEACON trial was discontinued after 9 months due to increased mortality and heart failure. Moreover, bardoxolone resulted in increased estimated GFR and urine ACR (29, 32), which was also confirmed in a recent trial of this drug in non-diabetic patients with Alport syndrome (CARDINAL trial) [57]. There has been a concern of increased intraglomerular pressure induced by bardoxolone, but its mechanisms remain unclear [58,59]. We speculate that bardoxolone upregulation of SGLT2 expression in RPTs might have played a role in these events, ultimately leading to glomerular hyperfiltration via tubulo-glomerular feedback and aggravated kidney injury. Indeed, the recent studies of Rush et al. [60] have reported that genetic activation of Nrf2 in *Keap1*^FA/FA^ mice or pharmacologic Nrf2 activation via bardoxolone analog increase proteinuria in several mouse models of chronic kidney disease. However, the question whether NRF2 activation may be harmful in T2D patients with CKD warrants further investigation.

## 5. Conclusions

Our present findings have demonstrated that genetic deletion of *Nrf2* can lead to the down-regulation of renal AGT, SGLT2, CD36, and FABP4 expression with subsequent decreases in SBP, FBG, and tubular lipid accumulation, which ultimately resulted in the attenuation of kidney injury in db/db *Nrf2* KO mice. Our results imply an important role for NRF2-mediated stimulation of AGT, SGLT2, CD36, and FABP4 expression in exacerbating renal injury in T2D. Our findings would indicate that selective targeting of renal NRF2 might be a novel approach for the prevention or treatment of diabetic kidney disease.

## Data Availability

All data sets that were generated or analyzed during the current study are included in the published article (and its online supplementary files) and are available to other research groups upon reasonable request.

## Supplementary Materials

The following supporting information can be downloaded at: https://www.mdpi.com/article/10.3390/antiox12091715/s1. Reference [61] is cited in the supplementary materials.

## References

[1] Gai Z., Wang T., Visentin M., Kullak-Ublick G.A., Fu X., Wang Z. (2019). Lipid Accumulation and Chronic Kidney Disease. Nutrients.

[2] Moorhead J.F., Chan M.K., El-Nahas M., Varghese Z. (1982). Lipid nephrotoxicity in chronic progressive glomerular and tubulo-interstitial disease. Lancet.

[3] Herman-Edelstein M., Scherzer P., Tobar A., Levi M., Gafter U. (2014). Altered renal lipid metabolism and renal lipid accumulation in human diabetic nephropathy. J. Lipid Res..

[4] Falkevall A., Mehlem A., Palombo I., Sahlgren B.H., Ebarasi L., He L., Ytterberg A.J., Olauson H., Axelsson J., Sundelin B. (2017). Reducing VEGF-B Signaling Ameliorates Renal Lipotoxicity and Protects against Diabetic Kidney Disease. Cell Metab..

[5] Mitrofanova A., Fontanella A.M., Merscher S., Fornoni A. (2020). Lipid deposition and metaflammation in diabetic kidney disease. Curr. Opin. Pharmacol..

[6] Yang X., Okamura D.M., Lu X., Chen Y., Moorhead J., Varghese Z., Ruan X.Z. (2017). CD36 in chronic kidney disease: Novel insights and therapeutic opportunities. Nat. Rev. Nephrol..

[7] Hao J.-W., Wang J., Guo H., Zhao Y.-Y., Sun H.-H., Li Y.-F., Lai X.-Y., Zhao N., Wang X., Xie C. (2020). CD36 facilitates fatty acid uptake by dynamic palmitoylation-regulated endocytosis. Nat. Commun..

[8] Hou Y., Wang Q., Han B., Chen Y., Qiao X., Wang L. (2021). CD36 promotes NLRP3 inflammasome activation via the mtROS pathway in renal tubular epithelial cells of diabetic kidneys. Cell Death Dis..

[9] Susztak K., Ciccone E., McCue P., Sharma K., Böttinger E.P. (2005). Multiple metabolic hits converge on CD36 as novel mediator of tubular epithelial apoptosis in diabetic nephropathy. PLoS Med..

[10] Kralisch S., Fasshauer M. (2013). Adipocyte fatty acid binding protein: A novel adipokine involved in the pathogenesis of metabolic and vascular disease?. Diabetologia.

[11] Yeung D.C., Xu A., Tso A.W., Chow W., Wat N.M., Fong C.H., Tam S., Sham P.C., Lam K.S. (2009). Circulating levels of adipocyte and epidermal fatty acid–binding proteins in relation to nephropathy staging and macrovascular complications in type 2 diabetic patients. Diabetes Care.

[12] Ghezzi C., Loo D.D.F., Wright E.M. (2018). Physiology of renal glucose handling via SGLT1, SGLT2 and GLUT2. Diabetologia.

[13] Brady J.A., Hallow K.M. (2017). Model-Based Evaluation of Proximal Sodium Reabsorption Through SGLT2 in Health and Diabetes and the Effect of Inhibition With Canagliflozin. J. Clin. Pharmacol..

[14] Osorio H., Coronel I., Arellano A., Franco M., Escalante B., Bautista R. (2012). Ursodeoxycholic acid decreases sodium-glucose cotransporter (SGLT2) expression and oxidative stress in the kidney of diabetic rats. Diabetes Res. Clin. Pract..

[15] Umino H., Hasegawa K., Minakuchi H., Muraoka H., Kawaguchi T., Kanda T., Tokuyama H., Wakino S., Itoh H. (2018). High Basolateral Glucose Increases Sodium-Glucose Cotransporter 2 and Reduces Sirtuin-1 in Renal Tubules through Glucose Transporter-2 Detection. Sci. Rep..

[16] Wang X.X., Levi J., Luo Y., Myakala K., Herman-Edelstein M., Qiu L., Wang D., Peng Y., Grenz A., Lucia S. (2017). SGLT2 Protein Expression Is Increased in Human Diabetic Nephropathy: SGLT2 protein inhibition decreases renal lipid accumulation, inflammation, and the development of nephropathy in diabetic mice. J. Biol. Chem..

[17] Zhao S., Lo C.-S., Miyata K.N., Ghosh A., Zhao X.-P., Chenier I., Cailhier J.-F., Ethier J., Lattouf J.-B., Filep J.G. (2021). Overexpression of Nrf2 in Renal Proximal Tubular Cells Stimulates Sodium–Glucose Cotransporter 2 Expression and Exacerbates Dysglycemia and Kidney Injury in Diabetic Mice. Diabetes.

[18] Wanner C., Inzucchi S.E., Lachin J.M., Fitchett D., Von Eynatten M., Mattheus M., Johansen O.E., Woerle H.J., Broedl U.C., Zinman B. (2016). Empagliflozin and Progression of Kidney Disease in Type 2 Diabetes. N. Engl. J. Med..

[19] Perkovic V., Jardine M.J., Neal B., Bompoint S., Heerspink H.J.L., Charytan D.M., Edwards R., Agarwal R., Bakris G., Bull S. (2019). Canagliflozin and Renal Outcomes in Type 2 Diabetes and Nephropathy. N. Engl. J. Med..

[20] Neal B., Perkovic V., Matthews D.R. (2017). Canagliflozin and Cardiovascular and Renal Events in Type 2 Diabetes. N. Engl. J. Med..

[21] Heerspink H.J.L., Stefánsson B.V., Correa-Rotter R., Chertow G.M., Greene T., Hou F.-F., Mann J.F.E., McMurray J.J.V., Lindberg M., Rossing P. (2020). Dapagliflozin in Patients with Chronic Kidney Disease. N. Engl. J. Med..

[22] Huang C.-C., Chou C.-A., Chen W.-Y., Yang J.-L., Lee W.-C., Chen J.-B., Lee C.-T., Li L.-C. (2021). Empagliflozin Ameliorates Free Fatty Acid Induced-Lipotoxicity in Renal Proximal Tubular Cells via the PPARγ/CD36 Pathway in Obese Mice. Int. J. Mol. Sci..

[23] Venugopal R., Jaiswal A.K. (1996). Nrf1 and Nrf2 positively and c-Fos and Fra1 negatively regulate the human antioxidant response element-mediated expression of NAD(P)H:quinone oxidoreductase_1_ gene. Proc. Natl. Acad. Sci. USA.

[24] Surh Y.-J., Kundu J.K., Na H.-K. (2008). Nrf2 as a master redox switch in turning on the cellular signaling involved in the induction of cytoprotective genes by some chemopreventive phytochemicals. Planta Medica.

[25] Zoja C., Corna D., Nava V., Locatelli M., Abbate M., Gaspari F., Carrara F., Sangalli F., Remuzzi G., Benigni A. (2013). Analogs of bardoxolone methyl worsen diabetic nephropathy in rats with additional adverse effects. Am. J. Physiol.-Ren. Physiol..

[26] Tan S.M., Sharma A., Stefanovic N., Yuen D.Y., Karagiannis T.C., Meyer C., Ward K.W., Cooper M.E., de Haan J.B. (2014). Derivative of bardoxolone methyl, dh404, in an inverse dose-dependent manner lessens diabetes-associated atherosclerosis and improves diabetic kidney disease. Diabetes.

[27] Saha P.K., Reddy V.T., Konopleva M., Andreeff M., Chan L. (2010). The triterpenoid 2-cyano-3,12-dioxooleana-1,9-dien-28-oic-acid methyl ester has potent anti-diabetic effects in diet-induced diabetic mice and Lepr(db/db) mice. J. Biol. Chem..

[28] Pergola P.E., Raskin P., Toto R.D., Meyer C.J., Huff J.W., Grossman E.B., Krauth M., Ruiz S., Audhya P., Christ-Schmidt H. (2011). Bardoxolone methyl and kidney function in CKD with type 2 diabetes. N. Engl. J. Med..

[29] Pergola P.E., Krauth M., Huff J.W., Ferguson D.A., Ruiz S., Meyer C.J., Warnock D.G. (2011). Effect of bardoxolone methyl on kidney function in patients with T2D and stage 3b–4 CKD. Am. J. Nephrol..

[30] Jiang T., Huang Z., Lin Y., Zhang Z., Fang D., Zhang D.D. (2010). The protective role of Nrf2 in streptozotocin-induced diabetic nephropathy. Diabetes.

[31] de Zeeuw D., Akizawa T., Audhya P., Bakris G.L., Chin M., Christ-Schmidt H., Goldsberry A., Houser M., Krauth M., Lambers Heerspink H.J. (2013). Bardoxolone methyl in type 2 diabetes and stage 4 chronic kidney disease. N. Engl. J. Med..

[32] de Zeeuw D., Akizawa T., Agarwal R., Audhya P., Bakris G.L., Chin M., Krauth M., Heerspink H.J.L., Meyer C.J., McMurray J.J. (2013). Rationale and Trial Design of Bardoxolone Methyl Evaluation in Patients with Chronic Kidney Disease and Type 2 Diabetes: The Occurrence of Renal Events (BEACON). Am. J. Nephrol..

[33] Zhao S., Ghosh A., Lo C.-S., Chenier I., Scholey J.W., Filep J.G., Ingelfinger J.R., Zhang S.-L., Chan J.S.D. (2018). Nrf2 Deficiency Upregulates Intrarenal Angiotensin-Converting Enzyme-2 and Angiotensin 1-7 Receptor Expression and Attenuates Hypertension and Nephropathy in Diabetic Mice. Endocrinology.

[34] Wakabayashi N., Dinkova-Kostova A.T., Holtzclaw W.D., Kang M.-I., Kobayashi A., Yamamoto M., Kensler T.W., Talalay P. (2004). Protection against electrophile and oxidant stress by induction of the phase 2 response: Fate of cysteines of the Keap1 sensor modified by inducers. Proc. Natl. Acad. Sci. USA.

[35] Abdo S., Lo C.-S., Chenier I., Shamsuyarova A., Filep J.G., Ingelfinger J.R., Zhang S.-L., Chan J.S.D. (2013). Heterogeneous nuclear ribonucleoproteins F and K mediate insulin inhibition of renal angiotensinogen gene expression and prevention of hypertension and kidney injury in diabetic mice. Diabetologia.

[36] Zhang S.-L., Chen X., Lecleric M., Henley N., Allidina A., Hallé J.P., Brunette M.-G., Filep J.G., Tang S.-S., Ingelfinger J.R. (2002). Hyperglycemia induces insulin resistance on angiotensinogen gene expression in diabetic rat kidney proximal tubular cells. J. Endocrinol..

[37] Gundersen H.J.G. (1988). The nucleator. J. Microsc..

[38] Weibel E.R. (1980). Numerical density: Shape and size of particles. Sterological Methods.

[39] Chen J., Chen J.-K., Conway E.M., Harris R.C. (2013). Survivin mediates renal proximal tubule recovery from AKI. J. Am. Soc. Nephrol..

[40] Schulte B.A., Spicer S.S. (1983). Histochemical evaluation of mouse and rat kidneys with lectin-horseradish peroxidase conjugates. Am. J. Anat..

[41] Ryan M.J., Johnson G., Kirk J., Fuerstenberg S.M., Zager R.A., Torok-Storb B. (1994). HK-2: An immortalized proximal tubule epithelial cell line from normal adult human kidney. Kidney Int..

[42] Lo C.-S., Miyata K.N., Zhao S., Ghosh A., Chang S.-Y., Chenier I., Filep J.G., Ingelfinger J.R., Zhang S.-L., Chan J.S.D. (2019). Tubular Deficiency of Heterogeneous Nuclear Ribonucleoprotein F Elevates Systolic Blood Pressure and Induces Glycosuria in Mice. Sci. Rep..

[43] Roche E., Buteau J., Aniento I., Reig J.A., Soria B., Prentki M. (1999). Palmitate and oleate induce the immediate-early response genes c-fos and nur-77 in the pancreatic beta-cell line INS-1. Diabetes.

[44] Lo C.-S., Shi Y., Chenier I., Ghosh A., Wu C.-H., Cailhier J.-F., Ethier J., Lattouf J.-B., Filep J.G., Ingelfinger J.R. (2017). Heterogeneous Nuclear Ribonucleoprotein F Stimulates Sirtuin-1 Gene Expression and Attenuates Nephropathy Progression in Diabetic Mice. Diabetes.

[45] Sharma K., McCue P., Dunn S.R. (2003). Diabetic kidney disease in thedb/dbmouse. Am. J. Physiol.-Ren. Physiol..

[46] Cherney D.Z., Perkins B.A., Soleymanlou N., Maione M., Lai V., Lee A., Fagan N.M., Woerle H.J., Johansen O.E., Broedl U.C. (2014). Renal hemodynamic effect of sodium-glucose cotransporter 2 inhibition in patients with type 1 diabetes mellitus. Circulation.

[47] Kidokoro K., Cherney D.Z., Bozovic A., Nagasu H., Satoh M., Kanda E., Sasaki T., Kashihara N. (2019). Evaluation of glomerular hemodynamic function by empagliflozin in diabetic mice using in vivo imaging. Circulation.

[48] Steinhausen M., Kucherer H., Parekh N., Weis S., Wiegman D.L., Wilhelm K.R. (1986). Angiotensin II control of the renal microcirculation: Effect of blockade by saralasin. Kidney Int..

[49] Remuzzi G., Perico N., Macia M., Ruggenenti P. (2005). The role of renin-angiotensin-aldosterone system in the progression of chronic kidney disease. Kidney Int..

[50] Abdo S., Shi Y., Otoukesh A., Ghosh A., Lo C.-S., Chenier I., Filep J.G., Ingelfinger J.R., Zhang S.L., Chan J.S. (2014). Catalase overexpression prevents nuclear factor erythroid 2–related factor 2 stimulation of renal angiotensinogen gene expression, hypertension, and kidney injury in diabetic mice. Diabetes.

[51] Guerrero-Hue M., Rayego-Mateos S., Vázquez-Carballo C., Palomino-Antolín A., García-Caballero C., Opazo-Rios L., Morgado-Pascual J.L., Herencia C., Mas S., Ortiz A. (2020). Protective role of Nrf2 in renal disease. Antioxidants.

[52] Yoh K., Hirayama A., Ishizaki K., Yamada A., Takeuchi M., Yamagishi S.-I., Morito N., Nakano T., Ojima M., Shimohata H. (2008). Hyperglycemia induces oxidative and nitrosative stress and increases renal functional impairment in Nrf2-deficient mice. Genes Cells.

[53] Liu Y., Uruno A., Saito R., Matsukawa N., Hishinuma E., Saigusa D., Liu H., Yamamoto M. (2022). Nrf2 deficiency deteriorates diabetic kidney disease in Akita model mice. Redox Biol..

[54] Itoh K., Chiba T., Takahashi S., Ishii T., Igarashi K., Katoh Y., Oyake T., Hayashi N., Satoh K., Hatayama I. (1997). An Nrf2/Small maf heterodimer mediates the induction of phase II detoxifying enzyme genes through antioxidant response elements. Biochem. Biophys. Res. Commun..

[55] Maruyama A., Tsukamoto S., Nishikawa K., Yoshida A., Harada N., Motojima K., Ishii T., Nakane A., Yamamoto M., Itoh K. (2008). Nrf2 regulates the alternative first exons of CD36 in macrophages through specific antioxidant response elements. Arch. Biochem. Biophys..

[56] Sussan T.E., Jun J., Thimmulappa R., Bedja D., Antero M., Gabrielson K.L., Polotsky V.Y., Biswal S. (2008). Disruption of Nrf2, a key inducer of antioxidant defenses, attenuates ApoE-mediated atherosclerosis in mice. PLoS ONE.

[57] Warady B.A., Pergola P.E., Agarwal R., Andreoli S., Appel G.B., Bangalore S., Block G.A., Chapman A.B., Chin M.P., Gibson K.L. (2022). Effects of Bardoxolone Methyl in Alport Syndrome. Clin. J. Am. Soc. Nephrol..

[58] Baigent C., Lennon R. (2018). Should we increase GFR with bardoxolone in alport syndrome?. J. Am. Soc. Nephrol..

[59] Quinlan C., Jayasinghe K. (2022). Bardoxolone methyl for Alport Syndrome: Opportunities and Challenges. Clin. J. Am. Soc. Nephrol..

[60] Rush B.M., Bondi C.D., Stocker S.D., Barry K.M., Small S.A., Ong J., Jobbagy S., Stolz D.B., Bastacky S.I., Chartoumpekis D.V. (2021). Genetic or pharmacologic Nrf2 activation increases proteinuria in chronic kidney disease in mice. Kidney Int..

[61] Wang L., Lei C., Zhang S.L., Roberts K.D., Tang S.S., Ingelfinger J.R., Chan J.S. (1998). Synergistic effect of dexamethasone and isoproterenol on the expression of angiotensinogen in immortalized rat proximal tubular cells. Kidney Int..

