# Supplementary material for: NRF2 Deficiency Attenuates Diabetic Kidney Disease in Db/Db Mice via Down-Regulation of Angiotensinogen, SGLT2, CD36, and FABP4 Expression and Lipid Accumulation in Renal Proximal Tubular Cells
Source: Antioxidants (Basel). 2023 Sep 4;12(9):1715. doi: 10.3390/antiox12091715 (PMC10525648; doi:10.3390/antiox12091715)
Supplement: Supplementary file 1 [file antioxidants-12-01715-s001.zip › antioxidants-2509930-supplementary.pdf]

Supplementary Figures

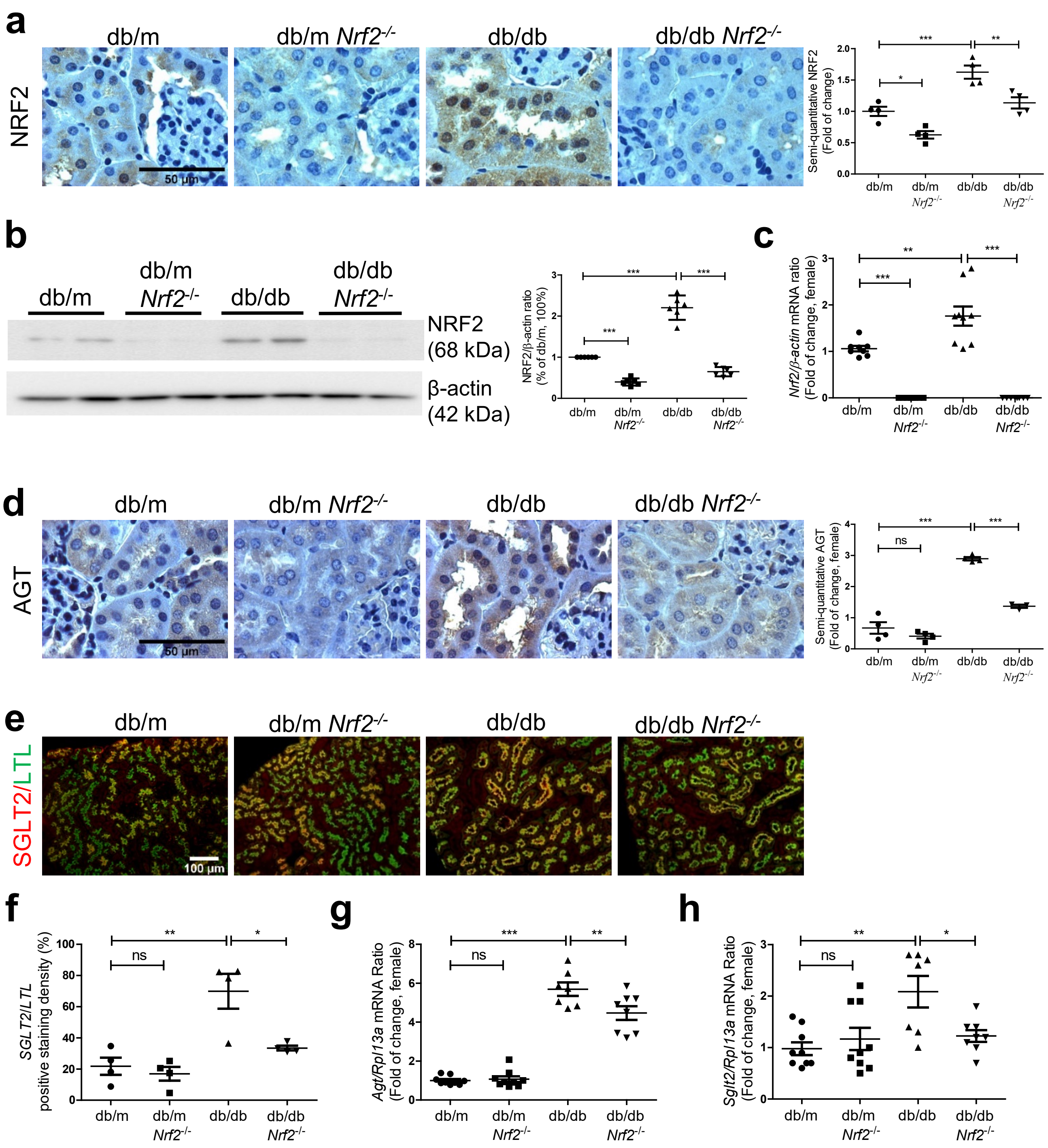

Figure S1 Nrf2, Keap1, Agt and Sglt2 expression in female mouse kidneys

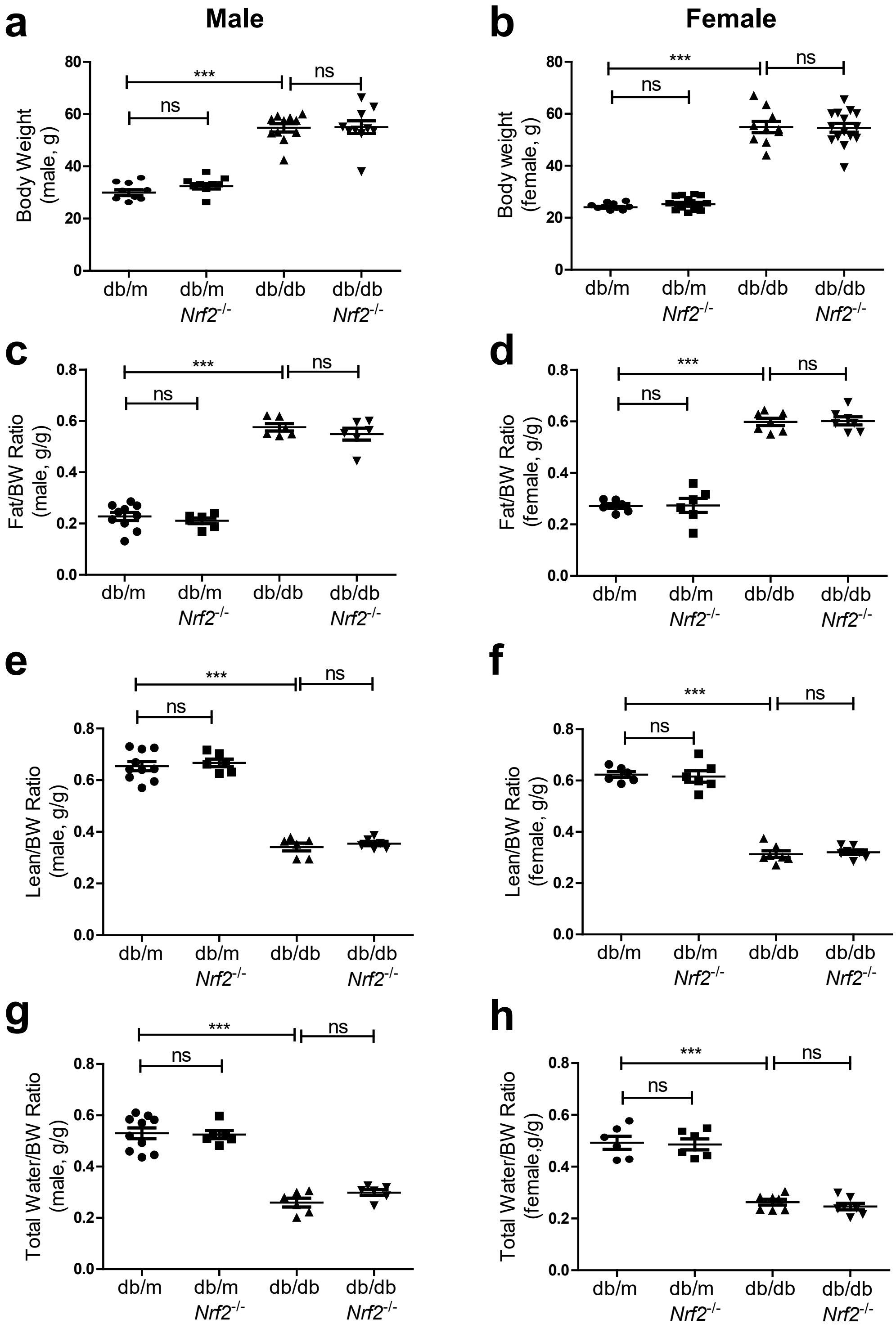

Figure S2 Physiological parameters in male and female mice at 16 weeks of age.

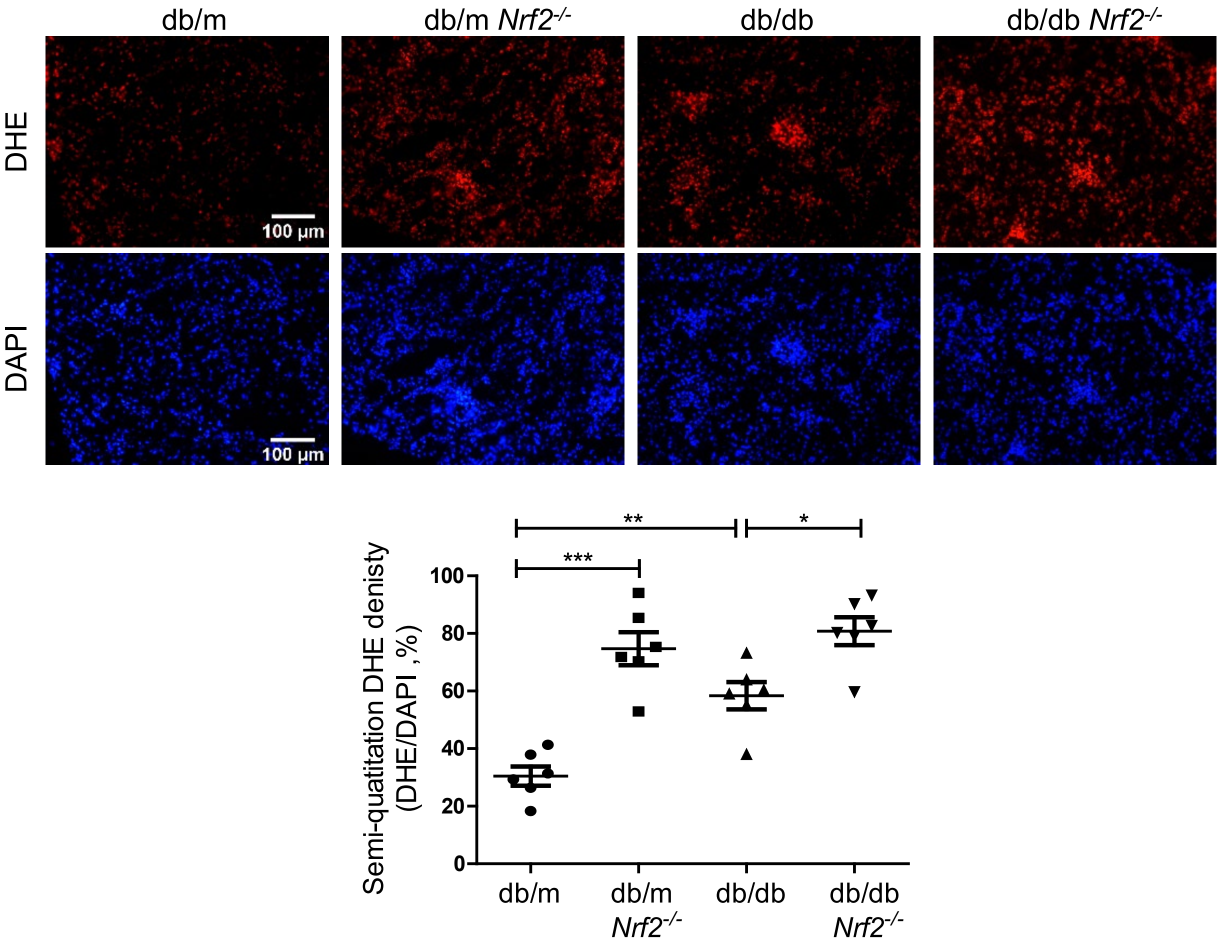

Figure S3 DHE and DAPI staining (x100) and semi-quantitation in kidney sections from male db/m, db/mNrf2 KO, db/db and db/dbNrf2 KO mouse kidneys at the age of 16weeks. Values are expressed as mean ± SEM, n=6 per group for staining. Statistics were done by one-way ANOVA, followed by Bonferroni post-hoc test.*$p<0.05$; **$p<0.01$; ***$p<0.005$ vs. db/m.

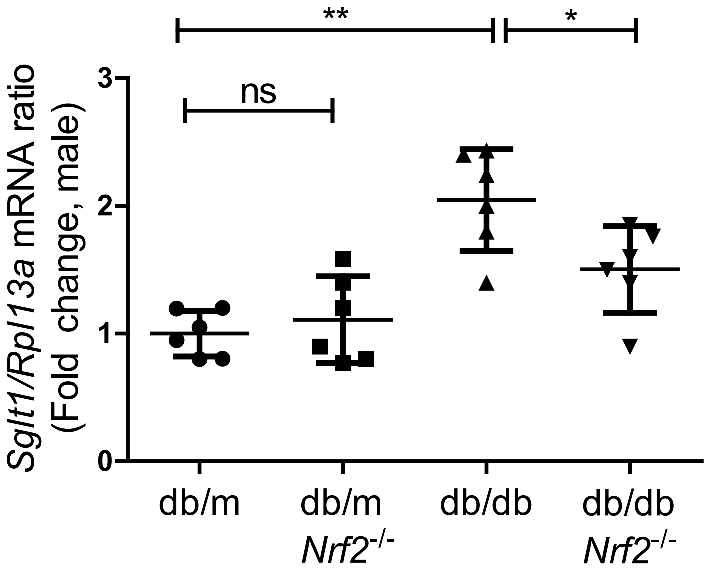

Figure S4. RT-qPCR analysis of Sglt1 mRNA levels in RPTs of male db/m, db/mNrf2 KO, db/db and db/dbNrf2 KO mice at age 16 weeks (n=6 per group). Statistics were done by one-way ANOVA, followed by Bonferroni post-hoc test. *$p<0.05$; **$p<0.01$; ***$p<0.005$.

Supplemental Table S1

Primary antibodies

Antibody (Host)	WB/IHC (Diultion)	Supplier	Cat#
NRF2 (mouse monoclonal)	WB(1:500);	Santa-Cruz	sc-365949
NRF2 (rabbit polyclona)	IHC (1:400)	Abcam	ab36113
KEAP1 (rabbit polyclonal)	WB(1:2000);	Abcam	ab66620
	IHC (1:400)		
AGT (rabbit polyclonal)	WB(1:2000);	Generated in our lab (1)	
	IHC (1:200)		
HO-1	IHC（1:400）<br>WB（1:2000）	ENZO	ADI-SPA-895-D
NOX4	IHC（1:400）	Abcam	ab109225
CATALASE	IHC（1:100）	Sigma-Aldrich	SAB5700937
SGLT2	IF（1:300）	Abcam	Ab85626
	WB（1:1000）	Alomone	AGT-032
β-Actin (mouse Monoclonal)	WB (1:10000)	Sigma-Aldrich	A5441
CD36（for mouse kidney）	IHC（1:200）	R&D Systems	AF2519
CD36（for human kidney）	IHC（1:400）	Proteintech	18836-1-AP
	WB（1:1000）		
FABP4（for mouse kidney）	IF（1:400）	R&D Systems	AF1443
FABP4（for human kidney）	IF（1:400）	R&D Systems	AF3150
LTL	IHC（1:200）	Invitrogen	L32480

(1) Wang L, Lei C, Zhang et al. Synergistic effect of dexamethasone and isoproterenol on the expression of angiotensinogen in immortalized rat proximal tubular cells. Kidney International 53:287-295, 1998.

Supplemental Table S2

Primers

Gene (Species)	Sense and Anti-Sense Primers	Reference Sequence
Primers for genotyping		
Nrf2 (mouse genotyping)	Common-S: GCCTGAGAGCTGTAGGCCC	NM_010902.3
	WT-AS: GGAATGGAAAATAGCTCCTGCC	
	Mutant-AS: GACAGTATCGGCCTCAGGAA	
Primers for RT-qPCR		
Nrf2 (mouse)	S: CGCCGCCTCACCTCTGCTGCCAGTAG	NM_010902.3
	AS: AGCTCATAATCCTTCTGTCG	
NRF2 (human)	S:ACACGGTCCACAGCTCATC	NM_006164.5
	AS:TGTCAATCAAATCCATGTCCTG	
Keap1 (mouse)	S: CATCCACCCTAAGGTCATGGA	NM_016679.4
	AS: GACAGGTTGAAGAACTCCTCC	
Agt (mouse)	S: CCACGCTCTCTGGATTTATC	NM_031144.3
	AS: ACAGACACCGAGATGCTGTT	
AGT (human)	S:AACTGGTGCTGCAAGGATCT	NM_000029.3
	AS:TCTCTCTCATCCGCTTCAAG	
Sglt2（mouse）	S: TTGGTGTTGGCTTGTGGTCTAT	NM_133254.4
	AS:ATGTTGCTGGCGAACAGAGA	
SGLT2（human）	S:CTGTTTGCACCCGTGTACCT	NM_003041
	AS:CCTGTCACCGTGTAAATCATGG	
RPL13a (mouse)	S:GCCCCACAAGACCAAGAGAG	NM_009438.5
	AS:TAGGCTTCAGCCGAACAACC	
RPL13a (human)	S:GCCCTACGACAAGAAAAAGCG	NM_012423.3
	AS:TACTTCCAGCCAACCTCGTGA	
CD36 (mouse)	S:GGAGCCATCTTTGAGCCTTCA	NM_001159558.1
	AS:GAACCAAACTGAGGAATGGATCT	
CD36 (human)	S:CTTTGGCTTAATGAGACTGGGAC	NM_001001547.3
	AS:GCAACAAACATCACCACACCA	
Fabp4 (mouse)	S:AAGGTGAAGAGCATCATAACCCT	NM_024406
	AS:TCACGCCTTTCATAACACATTCC	
HO-1(mouse)	S:GATAGAGCGCAACAAGCAGAA	NM_010442
	AS:CAGTGAGGCCCATACCAGAAG	
HO-1(human)	S:AAGACTGCGTTCCTGCTCAAC	NM_002133
	AS:AAAGCCCTACAGCAACTGTCG	
Catalase(mouse)	S:TGGCACACTTTGACAGAGAGC	NM_009804
	AS:CCTTTGCCTTGGAGTATCTGG	
NOX4(mouse)	S:GAAGGGGTTAAACACCTCTGC	NM_015760.5
	AS:ATGCTCTGCTTAAACACAATCCT	
SGLT1(mouse)	S:CACCGAGGGCTGACTCATTC	NM_019810
	AS:TGATCCGTACACCAGTACCAC	
